# Development of a Core Outcome Set in the Clinical Trials of Traditional Chinese Medicine for Stroke: A Study Protocol

**DOI:** 10.3389/fmed.2022.753138

**Published:** 2022-03-03

**Authors:** Ting Zhang, Xuechao Li, Liang Zhao, Jiaoyan Zhang, Jinhui Tian, Junhua Zhang

**Affiliations:** ^1^Evidence-Based Medicine Center, Tianjin University of Traditional Chinese Medicine, Tianjin, China; ^2^Clinical Research Center, Affiliated Hospital of Shaanxi University of Chinese Medicine, Xianyang, China; ^3^Evidence-Based Nursing Center, School of Nursing, Lanzhou University, Lanzhou, China; ^4^Evidence-Based Medicine Center, School of Basic Medical Sciences, Lanzhou University, Lanzhou, China; ^5^Key Laboratory of Evidence-Based Medicine and Knowledge Translation of Gansu Provinch, Lanzhou, China

**Keywords:** stroke, traditional Chinese medicine, core outcome set, methodology, study protocol

## Abstract

**Introduction:**

Stroke, an acute cerebrovascular disease, is mainly caused by the sudden rupture or occlusion of blood vessels, and is subdivided into ischemic stroke and hemorrhagic stroke. It has become the second leading cause of death worldwide. In Chinese clinical practice, traditional Chinese medicine (TCM)/Integrative Medicine has been widely used for the treatment of stroke. Numerous randomized controlled trials (RCTs) of TCM/Integrative Medicine for stroke have been conducted to improve the efficacy and safety outcomes. However, their conclusions should be treated with caution because of the methodological quality defects in the clinical research. Pervasive inconsistencies are present in the outcomes collected and reported across these studies, which may lead to the pooling of discrepant data and preclude meta-analysis. The issue could be addressed by developing a core outcome set (COS).

**Aim:**

The aim of this study is to develop a COS in the clinical trials of TCM/Integrative Medicine in the treatment of stroke.

**Method and Analysis:**

A steering group will be set up to organize and guide the development of the COS. The study contains three phases: (I) development of an initial outcome list covering all relevant outcomes, *via* two steps: (i) systematic reviews of outcomes for clinical trials of TCM/ Integrative Medicine for stroke; (ii) semi-structured interviews with patients suffering from stroke; (II) conduction of three round of Delphi surveys with different stakeholder groups to prioritize important outcomes; (III) integration of outcomes into a core outcome set by a consensus meeting.

**Ethics and Dissemination:**

This study has been granted by the Ethics Committee of Tianjin University of Traditional Chinese Medicine (TJUTCM-EC20210003). When the COS is completed, we will publish it in an appropriate journal to promote further widespread use.

**Registration:**

This study has been registered at the Core Outcome Measures in Effectiveness Trials initiative, COMET database (Registration #1678).

## Introduction

Stroke, an acute cerebrovascular disease, is caused by the sudden rupture or occlusion of blood vessels and is subdivided into ischemic stroke (87%) and hemorrhagic stroke (13%) (1). Its global prevalence in 2017 was 104.2 million, with ischemic stroke affecting 82.4 million and hemorrhagic stroke affecting 17.9 million people (2). Nowadays, stroke has become the second largest cause of death worldwide (5.5 million deaths), next only to ischemic heart disease (3). In China, stroke remains the leading cause of death and disability among adults, and the burden posed by it is equally severe (4, 5). The incidence of stroke in China in 2030 is expected to increase by ~50% when compared with that in 2010, which poses a huge economic and quality-of-life burden to the patients as well as the society at large (6).

Traditional Chinese medicine (TCM), which is based on the knowledge and practice accumulated over 2,000 years, is well-received all over the world because of its unique advantages in treating certain diseases (7, 8). Syndrome differentiation constitutes the treatment principle in TCM, wherein the severity and periodization of the disease are revealed in combination with the constitution of the patients. Four diagnostic methods, namely, tongue examination, smelling examination, inquisition, and palpation (pulse taking and abdominal examination) are used to determine the TCM syndrome. Based on the different syndromes of disease, Chinese herbs or TCM formulas are applied.

In Chinese clinical practice, in addition to the conventional methods, such as thrombolysis, antiplatelet therapy, early anticoagulation, and nerve protection, TCM/Integrative Medicine is used to treat stroke (9). Numerous clinical trials and systematic reviews have been conducted on TCM/Integrative Medicine for stroke to improve the efficacy and safety outcomes (7, 9–11). However, their conclusions should be treated with caution because of the methodological quality defects in the clinical research. Pervasive inconsistencies are present in the outcomes collected and reported across these studies, which may lead to the pooling of discrepant data and preclude meta-analysis (12). Furthermore, such inconsistencies compromise the value of clinical trials and result in a wastage of resources. Therefore, it is necessary to develop a core outcome set (COS) to ensure consistent outcomes in future clinical trials.

Core outcome set denotes the minimum results that should be measured and reported in the clinical trials in a specific area of healthcare to reduce the heterogeneity between the reported outcomes and strengthen the evidence synthesis value by lowering the risk bias of outcome reporting (13). In 2010, the Core Outcome Measures in Effectiveness Trials (COMET) initiative was founded, with a commitment to develop, implement, disseminate, and update the COS (14).

After searching the COMET database, three published COSs for stroke were identified. The COS of palliative care for stroke recommended that shared decision-making and quality of life are the most important outcome domains for future trials of palliative care in stroke (15). The Stroke Recovery and Rehabilitation Roundtable Consensus advised that the measurement standards and patient characteristics should be obtained in all future stroke recovery trials. As per the recommendation, the time of poststroke should be considered, the data should be aligned with the international classification of functioning and disability, and kinematic and kinetic movement quantification should be included (16). The other COS recommended that survival and disease control, acute complications, and patient-reported outcomes must be assessed in ischemic stroke and intracerebral hemorrhage stroke trials (17). The stakeholders involved with the three related COSs represented an international perspective and included patient-reported outcomes. The standardized outcome sets in two related COSs may be biased toward Western patient populations, lacking engagement with the Chinese clinical experts and the Chinese patients (15, 16). None of the related COSs involve any outcome related to TCM syndromes. The characteristics of the three published COSs are shown in Supplementary Material 1. In addition, we identified six registered COSs of TCM for stroke and compared their characteristics and gaps, which are shown in Supplementary Material 2.

To deal with the problems related to the heterogeneity between the outcomes reported in the trials and the potential outcome reporting bias, it is imperative to develop a COS for stroke that can be used in the clinical trials of TCM/Integrative Medicine. The perspectives of all stakeholders for this specific area will be contained in the COS. Outcomes related to TCM syndromes will be included in it, which will be achieved by reaching a consensus between the Chinese clinical experts and patients with stroke.

## Aim and Scope

### Aim

To develop a COS in the clinical trials of TCM/Integrative Medicine for stroke.

### Scope of COS

We have developed the scope of this COS based on the criteria recommended by COMET (18). The scope of this COS is as follows:

1. Health condition: patients with ischemic stroke and patients with hemorrhagic stroke (age ≥ 18 years), including acute phase, recovery phase, and sequelae phase (19), without other complications.
2. Interventions: TCM [Chinese herbs, herbal decoctions, Chinese patent medicine (CPM), and acupuncture] or Integrative Medicine.
3. Setting: randomized controlled trials (RCTs).

## Methods and Analysis

This study has been enrolled at the COMET database (registration #1678, available at https://www.comet-initiative.org/studies/details/1678). We will apply the Core Outcome Set Standards for Protocol Items (the COS-STAP Statement) in the protocol to report all the phases of this COS (20).

### Steering Group

A steering group, which included two TCM/Integrated Medicine experts of cerebrovascular diseases, a Western medicine expert of cerebrovascular diseases, and a methodologist will guide the development of this COS. This group will make decisions based on methods, such as determining the scope of COS, selecting the appropriate consensus methods, and reviewing this protocol.

### Patient and Public Involvement

Patients will be recruited to join the semi-structured interviews, Delphi survey, and consensus meetings.

### Design

**Figure 1:**
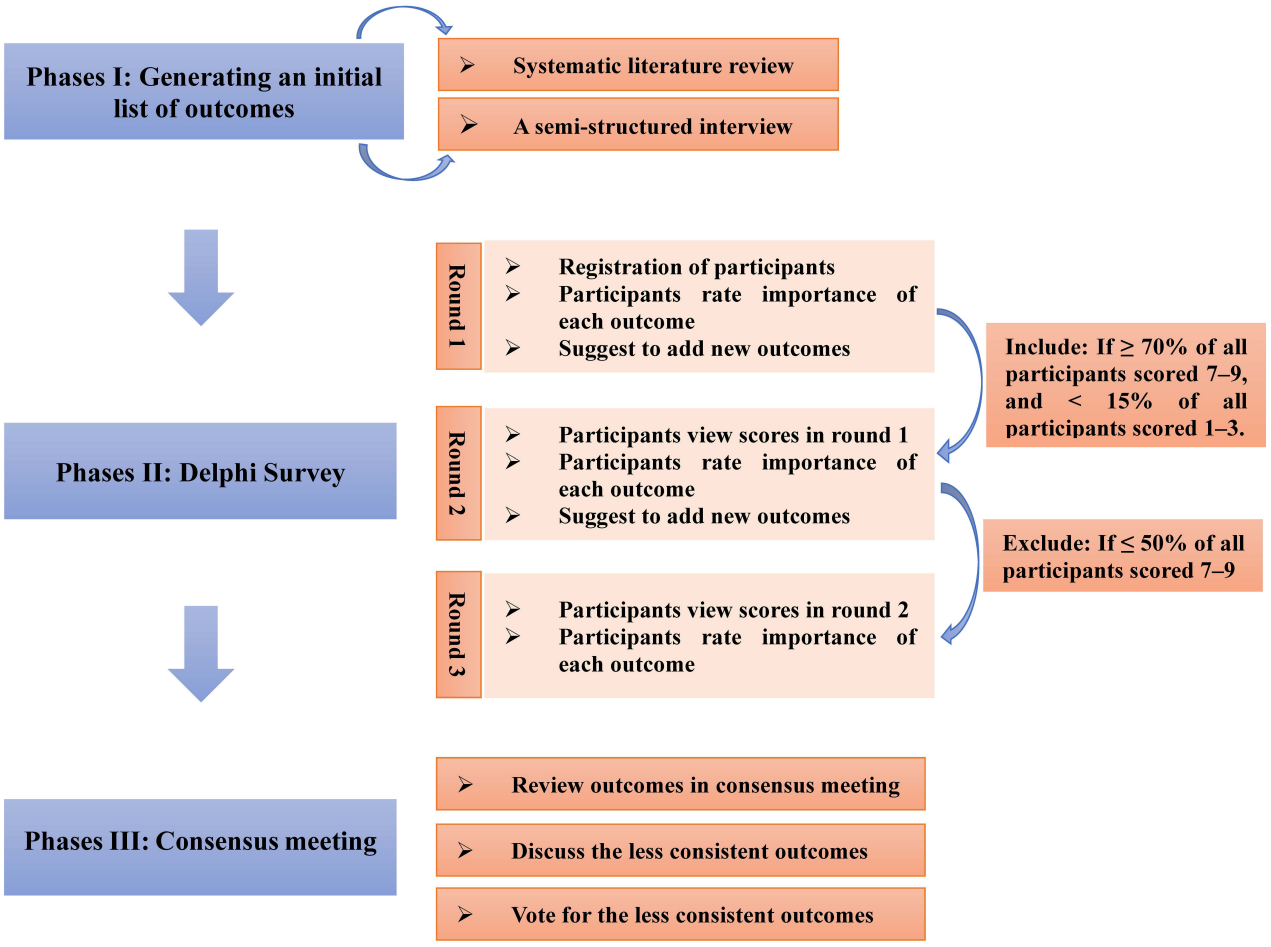
Key phases in process.

The COS will be developed in the following three phases (Figure 1):

Phases I: Developing an initial outcome list covering all possible relevant outcomes.

Phases II: Delphi survey with different stakeholder groups.

Phases III: Consensus meeting.

The details of the design process are as follows:

#### Phases I: Developing an Initial Outcome List Covering All Possible Relevant Outcomes

There are two steps involved in the development of an initial outcome list, namely, (i) systematic reviews and (ii) semi-structured interviews.

#### Step 1: Systematic Reviews of the Outcomes Reported in the Clinical Trials of TCM/Integrative Medicine for Stroke

##### Search Strategy

We will search three English databases and three Chinese databases, namely, PubMed, Embase, Cochrane Library, the China National Knowledge Infrastructure (CNKI), China Biology Medicine (CBM), and the Wanfang Data. The RCTs of stroke with TCM/Integ rative Medicine published from 2017 to 2021 will be included. The Language of publications will be restricted to English and Chinese only. The search strategies of three English databases are shown in Supplementary Material 3.

##### Eligibility Criteria

The inclusion and exclusion criteria for the systematic reviews are shown in Table 1.

**Table 1:** The inclusion and exclusion criteria for systematic reviews.

**Inclusion criteria**	**Exclusion criteria**
Patients with ischemic stroke, or hemorrhagic stroke (age ≥ 18 years), including the acute phase, recovery phase, or sequelae phase	Patients with other complications
TCM (Chinese herbs, herbal decoctions, Chinese patent medicine (CPM) and acupuncture) or integrative medicine	Rehabilitation, tuina, moxibustion, or exercise treatments such as taiji
Conventional western medicines or placebo	None
All outcomes reported in eligible RCTs	Outcomes evaluate mechanism or pharmacokinetics of drugs
Randomized controlled trials	Full-text cannot be obtained
RCTs were published in Chinese or English	

##### Study Selection

Two reviewers will screen the titles and abstracts of all records, independently, by using the Endnote X8 literature management software [Thomson Reuters (Scientific) LLC, Philadelphia, PA]. If any studies cannot be determined *via* the title and abstract, full-text reading will be implemented to identify them. Any disagreement will be resolved *via* discussion or consulting the steering group.

##### Data Extraction

Two reviewers will extract the data independently, including the name of the first author, sample size, age, sex, interventions, comparisons, and outcomes (including outcome name and definition, outcome measurement instrument, and outcome measurement timepoints). When the TCM syndromes are reported in the clinical trials of TCM/Integrated Medicine, the name and diagnostic criteria of TCM syndrome will also be extracted. In addition, we plan to use the method that has been used by a previous study to assess the quality of outcome in the eligible studies (21, 22). Any discrepancies will be resolved by mutual discussion or consulting the steering group. The items and scoring criteria are shown in Supplementary Material 4.

#### Step 2: Semi-Structured Interviews

##### Participant Selection

The opinion of the patient is an integral part of the COS development (23). We will recruit patients with stroke/caregivers to participate in the semi-structured interviews. The inclusion and exclusion criteria for the semi-structured interviews are shown in Table 2.

**Table 2:** The inclusion and exclusion criteria for the semi-structured interviews.

**Inclusion criteria**	**Exclusion criteria**
Patients with ischemic stroke, or hemorrhagic stroke (age ≥ 18 years), including the acute phase, recovery phase, or sequelae phase	Patients with a serious psychological or mental disease
Patients received TCM/integrated medicine treatment previously in neurology department pertaining to a tertiary hospital	
Caregivers who are taking care of patients with stroke	
Patients/caregivers voluntarily participated and signed informed consent	

##### Sampling Strategy

The purpose of the semi-structured interviews is to achieve “data saturation” (24). Based on the previous research experience, 30 patients were deemed sufficient to achieve saturation (22, 25). We will then recruit patients through snowball sampling method. In case of a new perspective in the final interview, the sample size will be increased.

##### Recruitment and Data Collection

We plan to approach potential patients in the inpatient ward of TCM-Affiliated First Hospital of the Tianjin University of Chinese Medicine and Affiliated Hospital of the Shaanxi University of Chinese Medicine. Two investigators who are trained in qualitative research methods will conduct the interview, and they will introduce the purpose and content of the interview to the participants. The patients will accordingly receive and read separate written information. The patients who agree to join the interview will provide their signed informed consent. Subsequently, each patient will be interviewed face-to-face for 20–30 min in the scientific research reception room of these hospitals. After obtaining the consent of the patients, the content of the interview will be recorded in audio. Simultaneously, we will obtain the demographic characteristics and drug application histories of interviewees through the electronic case systems of the hospitals. The information of the demographic characteristics and drug application histories of patients is shown in Supplementary Material 5.

The outline of the semi-structured interviews are as follows:

1. When were you diagnosed with a stroke, ischemic stroke, or hemorrhagic stroke?
2. What inconvenience or discomfort did you suffer from a stroke?
3. What treatments have you received for stroke?
4. What therapeutic effects do you hope to achieve?
5. What are the outcomes that you are most concerned about?

##### Data Analyses

We will analyze the results of the semi-structured interviews simultaneously with data collection. To achieve the outcomes mentioned in the interviews, the audio recording will be translated into words. Two investigators will read the translations word by word. The narrative explanations of the therapeutic effects of stroke and treatments on the lives of patients will be interpreted through the process of constant comparison to identify the outcomes that are important to patients (26) Then, two researchers will identify whether these outcomes are new. Any inconsistency will be discussed until reaching a consensus. After the review by the Steering Committee, the new outcomes will be added to the long list of outcomes.

##### Merging Outcomes and Grouping Outcomes Into Different Domains

When the systematic reviews and the semi-structured interviews are completed, the outcomes will be merged and grouped into different domains, based on the method recommended by previous COS studies (27, 28). This process will be conducted by two researchers independently. Any discrepancies will be resolved through discussion or by consulting the steering group. The details of the methods of merging outcomes and grouping them into domains are shown in Table 3.

**Table 3:** The details of the outcomes merging and grouping methods.

**Items**	**Methods**
1	For an English outcome, researcher will translate it into Chinese based on the terms formulated by the National Science and Technology Terminology Committee. If there is no corresponding term, it will be translated by two researchers
2	Composite outcomes will be separated into a single outcome
3	The overlapping outcomes will be merged into one based on the definition of the outcomes. Such as, effectiveness, efficacy, clinical efficacy, comprehensive efficacy, and therapeutic effect will be aggregated as “clinical efficiency”
3	Those outcomes without definition or measurement instrument will be dropped
4	Outcomes will be classified into different domains based on the taxonomy that has been developed by the COMET initiative (28)
5	The outcome domain of TCM characteristics will be added such as TCM syndrome scores

#### Phases II: Delphi Survey With Different Stakeholder Groups to Prioritize the Outcomes

Delphi survey is a structured and robust approach to gaining consensus, whereby different stakeholder groups will need to complete the sequential rounds of anonymized surveys (29). This process will avoid the need to select a few people for discussion or that the juniors agree with the opinions of the senior members (30). In this project, the survey will be fulfilled with the Delphi Manager, which is based on a web system to build and administer the Delphi surveys.

##### Stakeholder Selection

We will invite healthcare professionals [TCM clinical experts of cerebrovascular diseases, Western medicine clinical experts of cerebrovascular diseases, researchers, evidence-based medicine (EBM) methodologists, and journal editors], and patients (ischemic or hemorrhagic stroke) to join the three rounds of the Delphi survey. The inclusion and exclusion criteria for healthcare professionals in the Delphi survey are shown in Table 4. The inclusion and exclusion criteria for patients in the Delphi survey are shown in Table 5.

**Table 4:** The inclusion and exclusion criteria for healthcare professionals in the Delphi survey.

**Inclusion criteria**	**Exclusion criteria**
Clinical experts of TCM/integrated medicine, and western medicine of cerebrovascular diseases with over 5 years of work experience in tertiary hospitals and a master's degree or above. They will be selected from the China Association of Chinese Medicine (CACM)	None
Researchers (either first author or corresponding) have published articles regarding to stroke	
EBM methodologists will be selected from EBM center of Tianjin University of Traditional Chinese Medicine and EBM center of Lanzhou University	
There will be no restriction on the geographical area of experts	

**Table 5:** The inclusion and exclusion criteria for patients in the Delphi survey.

**Inclusion criteria**	**Exclusion criteria**
Patients with ischemic stroke, or hemorrhagic stroke (age ≥ 18 years), coming from the TCM-Affiliated First Hospital of Tianjin University of Chinese Medicine and Affiliated Hospital of Shaanxi University of Chinese Medicine, without restriction on gender	Patients with severe mental disease
Patients who have the ability of literacy and communication	
Patients who have signed the informed consent forms	

##### Delphi Sample Size

Until date, there has been no standard sample size calculation method in the Delphi survey in the development of COS studies (31), and it is a general practice to use previous studies as an indicator (32). Referring to the previous COS study, the number of stakeholders in the Delphi survey ranged from 12 to 174 (18). In this study, a total of six stakeholder groups will be included. We will use the snowball sampling method to recruit ~120 participants for the entire whole Delphi survey.

##### Consensus Standards

The consensus standards will be defined in accordance with the definition shared by the past research (33). (i) More than or equal to 70% participants scored the outcomes as 7–9, and <15% of the participants scored the outcomes as 1–3, and the corresponding outcomes will be included in the COS; (ii) ≤ 50% of the participants scored the outcome as 7–9, and these outcomes will be excluded.

##### Round 1 of the Delphi Survey

All candidate outcomes in different outcome domains will be included in the questionnaire of Round 1 of the Delphi survey. First, healthcare professionals will be invited to register in the Delphi Manager and then to score all the candidate items *via* an online survey. The scoring criteria will be based on a nine-point scoring system (18), with “1–3” representing that the outcome is not important for the COS, “4–6” signifying that the outcome is important but not critical for the COS, and “7–9” denoting that the outcome is critical for the COS. If the participants find it difficult to score for any outcome, they will be able to choose “unclear.” At the end of the survey, the participants will have the opportunity to add additional outcomes that they think are important but not included in the candidate outcome list.

In the inpatient ward or the outpatient department of TCM-Affiliated First Hospital of Tianjin University of Chinese Medicine and Affiliated Hospital of Shaanxi University of Chinese Medicine, two researchers will independently converse with the qualified patients having stroke to explain the contents of the survey, supply separate written information sheets, and ask whether they agree to participate in the three rounds of the Delphi survey. Those who agree to participate will sign an informed consent form. Subsequently, they will have to complete the printed questionnaire. If they have any questions, the researchers will answer them on time. The researchers and patients will make an appointment for the next round of questionnaires. The patients will be informed that they can withdraw at any point in time.

Round 1 of the Delphi survey will be planned in such a way that it is completed within 3 weeks. The healthcare professionals will be prompted to finish the survey by sending them an e-mail at the end of the 2nd week. To reduce attrition bias, if the response rate is <80% at the end of the 3rd week, the time for Round 1 of the Delphi survey will be prolonged.

##### Analysis of the Data From Round 1 of the Survey

Descriptive statistics will be applied to analyze the response results of the health professionals and the patients, separately. The distribution of the scores for each outcome will be calculated for the whole Delphi survey and each stakeholder group. If an outcome is scored as ≥4 by ≥70% of the participants in any stakeholder group who complete the questionnaire, it will be included in Round 2. Any new outcomes recommended by the participants in Round 1 of the survey will be included in Round 2.

##### Round 2 of the Delphi Survey

In Round 2 of the survey, an improved questionnaire will be sent to the healthcare professionals who have completed Round 1 of the survey. Additionally, the score of the participants and the score distribution of their own stakeholders in Round 1 will be presented. The participants need to re-score the outcomes within 3 weeks. At the end of the 2nd week, they will be reminded to complete the Delphi survey. For the survey of the patients, the same methods as in Round 1 will be used. If the response rate is <80%, this round will be left open for a longer duration.

##### Analysis of the Data From Round 2 of the Survey

As in Round 1, the score distribution of each outcome will be summarized separately for the whole Delphi survey and each stakeholder group. The outcomes that agree with the consensus standards will be directly included in the consensus meeting. The rest of the outcomes will be carried forward to Round 3.

##### Round 3 of the Delphi Survey

In Round 3 of the survey, the healthcare professionals will be selected based on their completion of Round 2 survey, and then they will be sent an e-mail containing the scores and the distribution of each outcome for each stakeholder group as well as the overall Delphi survey from Round 2. At this time, the participants will be required to re-score each outcome on a nine-point scale. Again, a period of 3 weeks will be allotted for Round 3. Furthermore, the participants will be reminded to finish the survey *via* an e-mail sent at the end of the 2nd week. For the survey of patients, the same methods as in Round 2 will be employed. To reduce attrition bias, the survey period will be prolonged, if the response rate is <80% at the time of reaching the deadline.

##### Analysis of the Data From Round 3 of the Survey

The analysis process will follow the same method as in the previous two rounds. The outcomes will be classified as consensus in, consensus out, and no consensus. In due consideration of the consensus results of the previous two rounds, score distribution and the consensus results for each outcome will be presented by groupwise as well as overall and will be used to structure the final consensus meeting. Moreover, the potential attrition bias will be calculated based on the average score given by the participants who complete or do not complete the three rounds of the Delphi survey. In this study, the Delphi Manager, which is a special Delphi online survey software developed by the COMET working group for COS research (34), will be used to conduct the online survey. When applying the system, the electronic questionnaire will be set up with reminders for the missing data. If the questionnaire is not fully completed, it cannot be submitted, which will ensure the integrity of the questionnaire and effectively avoid the missing data problem.

#### Phases III: Consensus Meeting

After completing the Delphi survey, a face-to-face consensus meeting will be held in China with key stakeholders to finalize the COS.

##### Participants

There is no standard method to calculate the sample size for the consensus meeting in the development of COS studies (30). Therefore, we will invite ~25 stakeholders to participate in the consensus meeting, including (a) healthcare professionals who have more than 10 years of work experience in tertiary hospitals (TCM and Western medicine clinical experts of cerebrovascular diseases); (b) patients who fulfill all Delphi surveys; (c) EBM methodologists; (d) researchers with a master's degree or above; (e) journal editors; and (f) patients with stroke.

##### Process

First, the results of each round survey will be reviewed by a consensus meeting of the stakeholders, who will decide whether the consensus criteria are met. Moreover, the members of the consensus meeting will discuss the less-consistent outcomes. Finally, the stakeholders will vote for each less-consistent outcome with a similar approach as the one used in the Delphi survey, which will be processed anonymously.

##### Consensus Definition

The definition for consensus will be based on a previous COS research (18). The detailed information of the consensus definition is shown in Table 6.

**Table 6:** The consensus definition.

**Consensus classification**	**Description**	**Definition**
Consensus in	The outcome should be included in the COS	≥70% of all participants scored 7–9, and <15% of all participants scored 1–3
Consensus out	The outcome should not be included in the COS	≤ 50% of all participants scored 7–9
No consensus	Uncertainty about the importance of outcome	Anything else

## Discussion

At present, three published COS studies are available for stroke (15–17), which have paid attention to shared decision-making and the quality-of-life outcome domains, survival and disease control outcomes, and patient-reported outcomes. However, discrepancies exist in the individual outcomes between the different COSs. Regarding the composition of stakeholders in COS studies, only one COS study included the Chinese experts; however, it too did not contain Chinese patients with stroke (17). The remaining two COS studies included neither Chinese experts nor Chinese patients (15, 16). In Chinese clinical practice, TCM/Integrated Medicine is one of the key measures for stroke. So far, there is no published COS study on TCM/Integrative Medicine for stroke. Therefore, it is very necessary to develop a COS for stroke that can be used in the clinical trials of TCM/Integrative Medicine.

The content of this COS is based on the Core Outcome Set-Standardized Protocol (COS-STAP) guidelines (20). The COS will have the following advantages: (i) the value of clinical trials of TCM/Integrative Medicine for stroke will be significantly improved by using the COS, which will reduce the wastage of research resources; (ii) it will be conducive to improve the abilities to explain the clinical findings, compare the results from different trials, and synthesize the best available evidence by using meta-analysis; (iii) the risk of bias of selective outcome reporting will be reduced by the COS; (iv) the perspectives of different stakeholders will be included in the process of developing the COS. However, in this study, the language was restricted to English and Chinese. The literature in other languages was not included, which may cause a potential language bias.

TCM/integrative medicine has many advantages in the treatment of stroke, and several clinical trials have been conducted and published. However, most of the outcomes from these studies do not provide the evidence for the clinical practice, mainly because of the unstandardized outcome reporting. Therefore, it is imperative to develop an appropriate COS.

## Author's Note

When the COS development is completed, it will be published in a suitable journal, exchanged at a national and international stroke conference, and disseminated on the Chinese Medicine Association and its WeChat public platform to promote a wide range of use.

## Ethics Statement

The studies involving human participants were reviewed and approved by the Ethics Committee of Tianjin University of Traditional Chinese Medicine (TJUTCM-EC20210003). The patients/participants provided their written informed consent to participate in this study.

## Author Contributions

TZ, JT, and JuZ: conceived the project. TZ: wrote the original draft. TZ, XL, LZ, and JiZ were responsible for conducting the systematic review. TZ and XL were responsible for the management of the clinical research. JuZ and JT: provided supervision on all the aspects of the project. All authors have read and approved the manuscript.

## Funding

This project was supported by funding from the Research and Innovation Project of the Tianjin University of Traditional Chinese Medicine in 2020 (YJSKC-20201005), the Research and Innovation Projects of graduate students in Tianjin in 2020 (2020YJSB190), and Tianjin Science and Technology Planning Project (20JCJQJC00120).

## Conflict of Interest

The authors declare that the research was conducted in the absence of any commercial or financial relationships that could be construed as a potential conflict of interest.

## Publisher's Note

All claims expressed in this article are solely those of the authors and do not necessarily represent those of their affiliated organizations, or those of the publisher, the editors and the reviewers. Any product that may be evaluated in this article, or claim that may be made by its manufacturer, is not guaranteed or endorsed by the publisher.
