# Supplementary material for: Development of a Core Outcome Set in the Clinical Trials of Traditional Chinese Medicine for Stroke: A Study Protocol
Source: Front Med (Lausanne). 2022 Mar 3;9:753138. doi: 10.3389/fmed.2022.753138 (PMC8927076; doi:10.3389/fmed.2022.753138)
Supplement: Supplementary file 1 [file Table_1.docx]

**Supplementary Material 1. The characteristics of the three published core outcome set (COS) for stroke.**

| **Study ID** | **Disease Name** | **Intervention** | **Study method** | **Study type** | **Stakeholders** | **Geographical location** |
| --- | --- | --- | --- | --- | --- | --- |
| Mason B 2021 | Stroke | Palliative  care | Delphi process, Literature review, Semi-structured discussion | COS for clinical trials or clinical research | Clinical experts, Consumers (caregivers, patients), Researchers | International  -Europe (UK) |
| Kwakkel G 2017 | Stroke | Rehabilitation | Consensus conference, Semi-structured discussion, Survey | COS for clinical trials or clinical research | Clinical experts, Governmental agencies, Journal editors, Methodologists, Researchers, Service providers, Statisticians | International  -Europe (UK)  -North America (USA, Canada)  -Oceania (Australia) |
| Salinas 2016 | Stroke | Any | Delphi process, Teleconferences | COS for practice,  COS for registry | Clinical experts, Consumers (patients) | International  -Europe (UK, Sweden, Netherlands)  -North America (USA, Canada)  -Oceania (Australia)  -Asia (China) |
