# Supplementary material for: Development of a Core Outcome Set in the Clinical Trials of Traditional Chinese Medicine for Stroke: A Study Protocol
Source: Front Med (Lausanne). 2022 Mar 3;9:753138. doi: 10.3389/fmed.2022.753138 (PMC8927076; doi:10.3389/fmed.2022.753138)
Supplement: Supplementary file 2 [file Table_2.docx]

**Supplementary Material 2. The characteristics and gaps of the related COS studies for traditional Chinese medicine registered in the COMET database.**

| **Author** | **Study ID** | **Disease Name** | **Intervention** | **Study method** | **Study type** | **Stakeholders** |
| --- | --- | --- | --- | --- | --- | --- |
| Zhengna | 1958 | Acute cerebral infarction, Stroke | Complementary and alternative medicine (CAM) therapy, Traditional Chinese Medicine | Systematic review, Interview, Delphi process, Consensus meeting | -COS for clinical trials or clinical research;  -COS for practice | Charities, Clinical experts, Conference participants, Consumers (patients) |
| Yunling Zhang | 1475 | Hypertensive cerebral hemorrhage, Stroke | Traditional Chinese Medicine | Systematic review, Interview, Delphi process, Consensus meeting | -COS for clinical trials or clinical research;  -COS for practice | Clinical experts, Conference participants, Consumers (caregivers), Consumers (patients), Journal editors, Patient/ support group representatives, Researchers, Statisticians |
| Yunling Zhang | 1472 | Acute cerebral infarction, Stroke | Traditional Chinese Medicine | Systematic review, Interview, Delphi process, Consensus meeting | -COS for clinical trials or clinical research;  -COS for practice | Clinical experts, Conference participants, Consumers (caregivers), Consumers (patients), Journal editors, Patient/ support group representatives, Researchers, Statisticians |
| Jianxiong Cai | 1286 | Stroke | Other, Traditional Chinese Medicine | Literature review, Interview, Delphi process, Consensus meeting | -COS for clinical trials or clinical research;  -COS for practice | Clinical experts, Consumers (patients), Ethicists, Journal editors, Methodologists, Patient/ support group representatives, Policy makers, Regulatory agency representatives, Researchers, Statisticians |
| Xinfeng Guo | 1282 | Ischemic stroke, Acute ischemic stroke, Stroke | Traditional Chinese Medicine | Systematic review, Interview, Delphi process, Consensus meeting | -COS for clinical trials or clinical research;  -COS for practice | Clinical experts, Conference participants, Consumers (caregivers), Consumers (patients), Journal editors, Methodologists, Patient/ support group representatives, Researchers, Statisticians |
| Ting Zhang | 1678 | Ischemic Stroke and Hemorrhagic Stroke (including acute phase, recovery phase and sequelae phase) | Traditional Chinese Medicine (TCM)/Integrative Medicine | Systematic review,  Semi-structured interview, Delphi survey,  Consensus meeting | -COS for clinical trials or clinical research;  -COS for practice | Clinical experts, Researchers, Journal editors, Methodologists, Patient/caregivers, Statisticians |
