# Supplementary material for: Development of a Core Outcome Set in the Clinical Trials of Traditional Chinese Medicine for Stroke: A Study Protocol
Source: Front Med (Lausanne). 2022 Mar 3;9:753138. doi: 10.3389/fmed.2022.753138 (PMC8927076; doi:10.3389/fmed.2022.753138)
Supplement: Supplementary file 3 [file Table_3.docx]

**Supplementary Material 3. The search strategies of three English databases.**

| 1. **PubMed** | |
| --- | --- |
| #1 | "Stroke"[Mesh] |
| #2 | Stroke [Title/Abstract] |
| #3 | #1 OR #2 |
| #4 | "Medicine, Chinese Traditional"[Mesh] OR "Drugs, Chinese Herbal"[Mesh] |
| #5 | "Traditional Chinese Medicine"[Title/Abstract] OR TCM[Title/Abstract] OR "Chinese herbal drug"[Title/Abstract] OR "Chinese patent drug"[Title/Abstract] OR "integrative medicine"[Title/Abstract] |
| #6 | #4 OR #5 |
| #7 | "Clinical Trials, Phase II as Topic"[Mesh] OR "Clinical Trials, Phase III as Topic"[Mesh] OR "Clinical Trials, Phase IV as Topic"[Mesh] OR "Controlled Clinical Trials as Topic"[Mesh] OR "Randomized Controlled Trials as Topic"[Mesh] OR "Intention to Treat Analysis"[Mesh] OR "Pragmatic Clinical Trials as Topic"[Mesh] |
| #8 | Clinical Trials, Phase II[Publication Type] OR "Clinical Trials, Phase III"[Publication Type] OR "Clinical Trials, Phase IV"[Publication Type] OR "Controlled Clinical Trials"[Publication Type] OR "Randomized Controlled Trials"[Publication Type] OR "Pragmatic Clinical Trials as Topic"[Publication Type] OR "Single-Blind Method"[Mesh] OR "Double-Blind Method"[Mesh] OR random*[Title/Abstract] OR blind*[Title/Abstract] OR singleblind*[Title/Abstract] OR doubleblind*[Title/Abstract] OR trebleblind*[Title/Abstract] OR tripleblind*[Title/Abstract] |
| #9 | #7 OR #8 |
| #10 | #3 AND #6 AND #9 |
| 1. **Cochrane Library** | |
| #1 | MeSH descriptor: [Stroke] explode all trees |
| #2 | (Stroke): ti,ab,kw |
| #3 | #1 OR #2 |
| #4 | (“Traditional Chinese Medicine”): ti,ab,kw OR (TCM): ti,ab,kw OR (“Chinese herbal drug”): ti,ab,kw OR (“Chinese patent drug”): ti,ab,kw OR (“integrative medicine”): ti,ab,kw |
| #5 | #3 AND #4 |
| 1. **Embase** | |
| #1 | stroke:ti,ab,kw |
| #2 | 'cerebrovascular accident'/exp |
| #3 | #1 OR #2 |
| #4 | 'chinese medicine'/exp |
| #5 | 'traditional chinese medicine': ti,ab,kw OR 'chinese herbal drug':ti,ab,kw OR 'chinese patent drug':ti,ab,kw OR 'integrative medicine':ti,ab,kw |
| #6 | #4 OR #5 |
| #7 | 'phase 2 clinical trial (topic)'/exp |
| #8 | 'phase 3 clinical trial (topic)'/exp |
| #9 | 'phase 4 clinical trial (topic)'/exp |
| #10 | 'controlled clinical trial (topic)'/exp |
| #11 | 'randomized controlled trial (topic)'/exp |
| #12 | 'intention to treat analysis'/exp |
| #13 | 'pragmatic trial'/exp |
| #14 | 'single blind procedure'/exp |
| #15 | 'double blind procedure'/exp |
| #16 | random*: ab,ti OR blind*:ab,ti OR 'single blind*':ab,ti OR 'double blind*':ab,ti OR 'treble blind*':ab,ti OR 'triple blind*':ab,ti |
| #17 | #7 OR #8 OR #9 OR #10 OR #11 OR #12 OR #13 OR #14 OR #15 OR #16 |
| #18 | #3 AND #6 AND #17 |
