# Supplementary material for: Development of a Core Outcome Set in the Clinical Trials of Traditional Chinese Medicine for Stroke: A Study Protocol
Source: Front Med (Lausanne). 2022 Mar 3;9:753138. doi: 10.3389/fmed.2022.753138 (PMC8927076; doi:10.3389/fmed.2022.753138)
Supplement: Supplementary file 4 [file Table_4.docx]

**Supplementary Material 4. The items and the scoring criteria for assessment the reporting quality of outcomes.**

| **No.** | **Items** | **Yes** | **No** |
| --- | --- | --- | --- |
| 1 | Is the primary outcome clearly stated? | 1 point | 0 point |
| 2 | Is the primary outcome clearly defined so that another researcher would be able to reproduce its measurement? Where appropriate, this should include a clear description of time points, the person measuring the outcome, how the outcome was measured and where the out-come was measured？ | 1 point | 0 point |
| 3 | Are the secondary outcomes clearly stated? | 1 point | 0 point |
| 4 | Are the secondary outcomes clearly defined? | 1 point | 0 point |
| 5 | Do the authors explain the use of the outcomes they have selected? | 1 point | 0 point |
| 6 | Are methods used to enhance the quality of outcome measurement (for example, repeated measurement, training) if appropriate? | 1 point | 0 point |
