# Supplementary material for: Development of a Core Outcome Set in the Clinical Trials of Traditional Chinese Medicine for Stroke: A Study Protocol
Source: Front Med (Lausanne). 2022 Mar 3;9:753138. doi: 10.3389/fmed.2022.753138 (PMC8927076; doi:10.3389/fmed.2022.753138)
Supplement: Supplementary file 5 [file Table_5.docx]

**Supplementary Material 5. The information of the demographic characteristics and drug application histories of patients.**

| **Items** | **Patients 1** | **Patients 2** | **……** | **Patients 30** |
| --- | --- | --- | --- | --- |
| **Basic information** |  |  |  |  |
| Hospital ID |  |  |  |  |
| Age (year) |  |  |  |  |
| Sex (Male/Female) |  |  |  |  |
| Address |  |  |  |  |
| **Type and Phase of Stroke** |  |  |  |  |
| Ischemic Stroke |  |  |  |  |
| Acute phase |  |  |  |  |
| Recovery phase |  |  |  |  |
| Sequelae phase |  |  |  |  |
| Hemorrhagic Stroke |  |  |  |  |
| Acute phase |  |  |  |  |
| Recovery phase |  |  |  |  |
| Sequelae phase |  |  |  |  |
| **Drug application histories** |  |  |  |  |
| Thrombolysis |  |  |  |  |
| Antiplatelet Therapy |  |  |  |  |
| Early Anticoagulation |  |  |  |  |
| Nerve Protection |  |  |  |  |
| Traditional Chinese Medicine |  |  |  |  |
| Others |  |  |  |  |
